# Improved Posterolateral Lumbar Spinal Fusion Using a Biomimetic, Nanocomposite Scaffold Augmented by Autologous Platelet-Rich Plasma

**DOI:** 10.3389/fbioe.2021.622099

**Published:** 2021-08-16

**Authors:** Jeffrey L. Van Eps, Joseph S. Fernandez-Moure, Fernando J. Cabrera, Francesca Taraballi, Francesca Paradiso, Silvia Minardi, Xin Wang, Bayan Aghdasi, Ennio Tasciotti, Bradley K. Weiner

**Affiliations:** ^1^ Department of Surgery, University of Texas Health Science Center, McGovern Medical School, Houston, TX, United States; ^2^ Department of Surgery, Division of Trauma, Acute and Critical Care Surgery, Duke University Medical Center, Durham, NC, United States; ^3^ Michael E. DeBakey Department of Surgery, Baylor College of Medicine, Houston, TX, United States; ^4^ Center for Musculoskeletal Regeneration, Houston Methodist Academic Institute, Houston Methodist Research Institute, Houston, TX, United States; ^5^ Reproductive Biology and Gynaecological Oncology Group, Swansea University Medical School, Singleton Park, Swansea, United Kingdom; ^6^ Department of Orthopedic Surgery, Houston Methodist Hospital, Houston, TX, United States; ^7^ Center for Biomimetic Medicine, Houston Methodist Research Institute, Houston, TX, United States; ^8^ Sutter Gold Medical Foundation, Stockton, CA, United States; ^9^ IRCCS San Raffaele Hospital, Rome, Italy; ^10^ 3R Biotech, Milan, Italy; ^11^ Weill Cornell Medical College, Cornell University, New York, NY, United States

**Keywords:** spinal fusion, platelet-rich plasma, nanomaterials, biomaterials, scaffold, biomimicry, tissue engineering, bone regeneration

## Abstract

Remodeling of the human bony skeleton is constantly occurring with up to 10% annual bone volume turnover from osteoclastic and osteoblastic activity. A shift toward resorption can result in osteoporosis and pathologic fractures, while a shift toward deposition is required after traumatic, or surgical injury. Spinal fusion represents one such state, requiring a substantial regenerative response to immobilize adjacent vertebrae through bony union. Autologous bone grafts were used extensively prior to the advent of advanced therapeutics incorporating exogenous growth factors and biomaterials. Besides cost constraints, these applications have demonstrated patient safety concerns. This study evaluated the regenerative ability of a nanostructured, magnesium-doped, hydroxyapatite/type I collagen scaffold (MHA/Coll) augmented by autologous platelet-rich plasma (PRP) in an orthotopic model of posterolateral lumbar spinal fusion. After bilateral decortication, rabbits received either the scaffold alone (Group 1) or scaffold with PRP (Group 2) to the anatomic right side. Bone regeneration and fusion success compared to internal control were assessed by DynaCT with 3-D reconstruction at 2, 4, and 6 weeks postoperatively followed by comparative osteogenic gene expression and representative histopathology. Both groups formed significantly more new bone volume than control, and Group 2 subjects produced significantly more trabecular and cortical bone than Group 1 subjects. Successful fusion was seen in one Group 1 animal (12.5%) and 6/8 Group 2 animals (75%). This enhanced effect by autologous PRP treatment appears to occur *via* astounding upregulation of key osteogenic genes. Both groups demonstrated significant gene upregulation compared to vertebral bone controls for all genes. Group 1 averaged 2.21-fold upregulation of RUNX2 gene, 3.20-fold upregulation of SPARC gene, and 3.67-fold upregulation of SPP1 gene. Depending on anatomical subgroup (cranial, mid, caudal scaffold portions), Group 2 had significantly higher average expression of all genes than both control and Group 1–RUNX2 (8.23–19.74 fold), SPARC (18.67–55.44 fold), and SPP1 (46.09–90.65 fold). Our data collectively demonstrate the osteoinductive nature of a nanostructured MHA/Coll scaffold, a beneficial effect of augmentation with autologous PRP, and an ability to achieve clinical fusion when applied together in an orthotopic model. This has implications both for future study and biomedical innovation of bone-forming therapeutics.

## Introduction

A host of medical bone-forming therapeutic applications have emerged to treat pathological conditions in America’s aging and enlarging population, including osteoporosis, fracture healing, and spinal fusion (Johnson et al., 2000; Mundy, 2002). Despite this, no reliable osteogenic agent has been developed and applied clinically with satisfactory cost, efficacy, and safety (Rodan et al., 2000; Yoon and Boden, 2002). Arthritis and degenerative disorders of the cervical and lumbar spine are routinely treated surgically with an arthrodesis procedure designed to produce fusion between adjacent vertebral levels. Conditions necessitating spinal fusion range from degenerative disease to instability from trauma, to spinal pseudoarthroses, or tumors (Sengupta and Herkowitz, 2003; Verlaan et al., 2004; Deyo et al., 2005). Current surgical techniques to accomplish mechanical stabilization across a diseased spinal level commonly utilize a combination of permanent synthetic hardware such as plates, screws, and cages with either autologous bone or bioprosthetic products.

Recombinant Human Bone Morphogenetic Protein-2 (rhBMP-2) was approved for single-level anterior lumbar interbody fusion (ALIF) in 2002 by the US Food and Drug Administration (FDA) (Xiong et al., 2013). Use of rhBMP-2 increased significantly thereafter, extending beyond approved indications to off-label orthopedic usage, and was commonly used to augment posterior lumbar spinal fusion (PLSF) and cervical spine fusion (Mitka, 2011). Although fusion efficacy has never been an issue with rhBMP-2, perhaps due at least in part to the supratherapeutic dosages employed, an under-reporting of side effects associated with its use was demonstrated upon critical data review (Weiner and Walker, 2003; Carragee et al., 2011) and confirmed by the Yale Open Data Access (YODA) project (Krumholz and Waldstreicher, 2016). Such adverse reactions include seroma formation, vertebral osteolysis, ectopic bone formation, retrograde ejaculation, and carcinogenicity (Carragee et al., 2012). The FDA issued a Public Health Notification warning for rhBMP-2 use in the wake of reports of airway edema and respiratory distress associated with its off-label cervical spine applications (Smucker et al., 2006; Carragee et al., 2011). Synthetic or exogenous growth factors like BMP are further limited by their proclivity for degradation or enzymatic deactivation after delivery, short physiologic half-life, and dependence on a finely-tuned carrier mechanism to avoid burst release (De Witte et al., 2018). As such, ongoing research has pursued a plausible alternative to rhBMP-2, but no suitable replacement with an enhanced safety profile and comparable efficacy has yet emerged. Successful development of alternative implantable therapeutics requires improving upon the rudimentary biomaterials (e.g., simplistic collagen sponges) employed or introducing novel, more efficient osteogenic compounds to decrease the requisite dosing to a safer therapeutic window. A plethora of new synthetic bioactive compounds are currently under investigation with a breadth that is, beyond the scope of this discussion (Ho-Shui-Ling et al., 2018). Though promising, such modalities require validation and safety assessment in well-designed human clinical trials.

Platelet-rich plasma (PRP) is most simply defined as a higher than normal concentration of platelets suspended within a volume of remaining platelet-poor plasma (PPP). Activated platelets within PRP not only provide a useful preliminary matrix for cellular population but they also release a host of chemokines and bioactive factors from prepackaged alpha granules that are effective in recruiting cells such as mesenchymal stromal cells (MSC) and fibroblasts to the site of injury and stimulating their subsequent proliferation and biosynthetic activity (Fernandez-Moure et al., 2017a). In prior work, we have fully characterized PRP and demonstrated its capability of inducing significant migration and proliferation of mesenchymal stem cells (MSC) (Murphy et al., 2012). The presence of pro-angiogenic factors such as vascular endothelial growth factor (VEGF) and platelet-derived growth factor (PDGF) along with the fibroblast-stimulating activity of transforming growth factor beta (TGF-β), make PRP an ideal therapy to promote soft tissue wound healing, which we have verified in multiple studies of augmented ventral hernia repair in rodents (Fernandez-Moure et al., 2015; Van Eps et al., 2016; Fernandez-Moure et al., 2017b; Van Eps et al., 2019). The TGF-β superfamily of growth factors is well known to be osteogenic, making the TGF-releasing ability of PRP specifically attractive as a candidate to enhance bone growth (Marx et al., 1998; Ramoshebi et al., 2002). PRP has been implemented successfully as an FDA-approved treatment modality for decades by orthopedic, oromaxillofacial, and plastic surgeons for purposes of implant ingrowth, dermal filling/contracture, and joint tendonopathies (Arora et al., 2009; Fernandez-Moure et al., 2017a). Although there exists an increasing focus on synthetic moieties in recent bioengineering, there is a concurrent clinical emphasis on the future of “personalized” or “precision” medicine whereby an individual’s unique genomic signature is used to tailor a therapeutic drug or implantable device for improved effect (Dugger et al., 2018; Sun et al., 2019). Autologous cell-based approaches have been featured in early forms of such novel platforms and are attractive for the obviated concern over donor-recipient compatibility and perceived ease of overcoming regulatory hurdles to clinical translation.

The gold standard substance employed to augment the success rate and quality of fusion has been autogenous iliac crest bone autograft (ICBG) (Aghdasi et al., 2013) harvested *via* separate incision site(s) at the time of surgery. As with any additional surgical procedure, utilizing ICBG carries inherent added risks and disadvantages, including increased blood loss, additional sites of mobility-limiting pain, and increased costs associated with longer operating times and hospital stays. Additionally, donor site morbidity from complications such as chronic pain or wound infection, and issues with both ICBG quality in smokers or insufficient graft quantity in multi-level fusions, have motivated the quest for alternative therapeutic interventions to augment fusion using biosynthetic and biopolymer grafting substitutes (Vaccaro, 2002; Di Martino et al., 2005; Rihn et al., 2010).

Several innovative biomaterial strategies are being investigated and show significant early potential, including functional surface modification, nanoparticle controlled drug release, and biohybrid approaches that include precellularization (Armentano et al., 2011; Devgan and Sidhu, 2019; Christy et al., 2020).

Bone is a natural composite material, mostly consisting of the calcium phosphate “hydroxyapatite” (HA) and type I collagen (Lyons et al., 2020). For this reason, both HA and collagen have been extensively used in orthopedic surgery, mostly as powders and sponges, respectively (Lyons et al., 2020). Numerous HA/collagen composites have also been proposed and tested, but all current formulations used in clinical practice are significantly lacking in osteoinductivity (Driscoll et al., 2020), still requiring the combination with ICBG and/or rhBMP-2 (Nickoli and Hsu, 2014). Nanostructured bioceramics and biocomposites have become increasingly attractive due to their ability to mimic the chemical-physical and morphological cues of bone at the nanoscale (Uskoković, 2015). Among these, nanostructured bio-hybrid composites offer novel capabilities to stimulate and enhance the bone regenerative process (Avitabile et al., 2020). Toward this end, we recently developed a biomimetic composite scaffold recapitulating the human trabecular bone niche at the nanoscale that proved effective at promoting osteogenesis in both an ectopic (Minardi et al., 2015) and orthotopic (Minardi et al., 2019) model of bone regeneration in the rabbit without the use of any biologics.

Although PRP has been used clinically since the 1980s and there exists a sizable body of literature supporting its utility in augmenting wound healing, diminishing pain and inflammation, and promoting tissue regeneration, conflicting reports regarding its efficacy to augment bone regeneration applications have left clinicians and scientists without a clear consensus (Hokugo et al., 2005; Albanese et al., 2013; Cinotti et al., 2013; Elder et al., 2015; Liu et al., 2017). One impetus for our study was to help answer once and for all whether autologous PRP has a role in improving bone regeneration platforms. For our purposes, PRP served as an easily attainable, surrogate source of growth factors for use on our novel, composite scaffold. The following study aims to contribute to the discovery and use of novel osteogenic therapeutics and test the *in vivo* osteogenic potential of a nanocomposite, multiphase scaffold when used alone or in concert with autologous platelet-rich plasma (PRP). Our hypothesis was that the biohybrid use of our novel nanocomposite scaffold with autologous PRP would be sufficient to induce bridging osteogenesis and fusion in an orthotopic rabbit model of lumbar spinal fusion.

## Materials and Methods

### Biohybrid Scaffold Synthesis

MHA/Coll was fabricated through a bioinspired mineralization process, as extensively described elsewhere (Minardi et al., 2015; Minardi et al., 2019; Avitabile et al., 2020; Mondragón et al., 2020). Briefly, bovine type I collagen (Viscofan Collagen United States Inc.) was dissolved in an aqueous acetic buffer solution (pH 3.5) at a concentration of 10 mg/ml. An aqueous solution of H_3_PO_4_ was added to the acetic collagen suspension and dropped into a basic solution of Ca(OH)_2_ and MgCl_2_·6H_2_O, all at equal 1:1 ratio of acetic collagen gel weight (40 g) to molar (40 mM) solution. The resulting mineralized collagen slurry was crosslinked in an aqueous 2.5 mM solution of 1,4-butanediol diglycidyl ether (BDDGE). The slurry was finally poured in plastic cylindrical molds (4 cm × 1 cm) and freeze-dried through an optimized freezing-heating ramp: the materials were frozen from +20°C to −20°C over 3 h, followed by reheating to +20°C over 5 h, under vacuum (20 mTorr).

### Scaffold Characterization and Biomimicry

Prior to implantation, MHA/Coll were fully characterized as previously reported (Minardi et al., 2015; Minardi et al., 2019). Characterization modalities included: scanning electron microscopy (SEM) and confocal fluorescent imaging after PRP-staining to evaluate overall morphology/topography with or without PRP, Fourier-transformed infrared spectroscopy (FTIR) to evaluate the chemical interactions between the functional groups of the mineral phase and organic template, and universal machine compression testing to distinguish mechanical properties. Briefly, empty MHA/Coll scaffolds, and MHA/Coll scaffolds with platelets alone or treated with 10% calcium chloride solution were dehydrated by graded ethanol solutions (30, 50, 75, 85, and 95% for 2 h each) and placed overnight in a dryer at room temperature before being coated by 12 nm of Pt/Pl for scanning electron microscopy (SEM; Nova NanoSEM 230, FEI, Hillsboro, OR, http://www.fei.com). PRP was isolated by the technique described below in detail for use in confocal microscopy. Pre-staining was performed of PRP alone or after treatment with 10% calcium chloride solution using PKH26 Red Fluorescent Cell Linker Kit for General Cell Membrane Labeling (Sigma Aldrich) before PRP seeding on MHA/Coll scaffolds. Imaging was performed using a Nikon A1 Confocal Imaging System. Collagen fiber autofluorescence emission was recorded in the DAPI channel. For FTIR analysis, samples were analyzed in ATR mode at 4 cm^−1^ resolution 256 times over the range of 500–2,000 cm^−1^ using a Nicolet 6,700 spectrometer. The ATR/FTIR spectra were reported after background subtraction, baseline correction and binomial smoothing (9 points). For compression analysis, empty MHA/Coll scaffolds, MHA/Coll scaffolds with platelets alone, and MHA/Coll scaffolds with platelets treated with 10% calcium chloride solution were loaded on a UniVert Mechanical Test System. A Load Cell of 10 N was calibrated and used to perform a compression test with stretch magnitude of 50% and a stretch duration of 60 s. The machine was setup with an appropriate load of 0.1 N specimens and a cross speed of 1 mm/min between two steel plates up to a strain level of approximately 50%. A minimum of three samples was used for each test.

### PRP Isolation, Quantification, and Activation

To obtain PRP for use in our study, 10–12cc of whole blood was harvested prior to surgery from each animal subject of Group 2 *via* standard auricular venipuncture into collection vials containing acid citrate dextrose (ACD) anticoagulant. A double centrifugation technique similar to that previously reported was used to isolate the PRP (Alsousou et al., 2009; Foster et al., 2009; Fernandez-Moure et al., 2015). Whole blood was spun initially at 200 g for 15 min to isolate the plasma fractions. The red blood cell (RBC) and buffy coat components were carefully removed manually by aspirating with a pipette and the remaining plasma was centrifuged a second time at 1600 g for 10 min. This second spin separates the platelet pellet from residual platelet-poor plasma (PPP). Following the manufacturer’s instructions, a Multisizer Coulter Counter (Beckman Coulter, Pasadena, CA) was used to quantify the platelets, which were appropriately diluted to prepare a strictly standardized final dose concentration of 1 × 10^6^ platelets per microliter of plasma in the therapeutic PRP delivered. Preoperative absorbency testing of our material revealed that 2 ml of aqueous solution is required to saturate the standardized 2.36 cm^3^ scaffold. The average platelet yield from each animal was limiting, at less than the 2 × 10^9^ required for a 2 ml volume of PRP at the above standardized concentration. Thus, a total of 1 × 10^9^ total platelets were diluted in 2 ml of PPP, for an effective dose concentration of 5 × 10^5^ platelets/microliter applied to each experimental scaffold. Group 1 animals received an identically sized MHA/Coll scaffold soaked with sterile phosphate buffered saline (PBS). A myriad of factors encompassing both extrinsic forces or chemicals and inherent factors within a traumatic/surgical wound itself are known to activate PRP, such as exposed tissue factor, type I collagen, shear forces and even platelet coagulation itself (Alberio et al., 2000; Kamath et al., 2001; Ruggeri, 2002). To ensure full release of the platelets’ alpha granule growth factor cargo, PRP activation occurred *via* a mixture of 10% calcium chloride solution and bovine thrombin (1000 U/mL, Sigma-Aldrich, St. Louis, MO) as previously reported (Everts et al., 2006; Araki et al., 2012; Fernandez-Moure et al., 2015).

### Study Design

**FIGURE 1:**
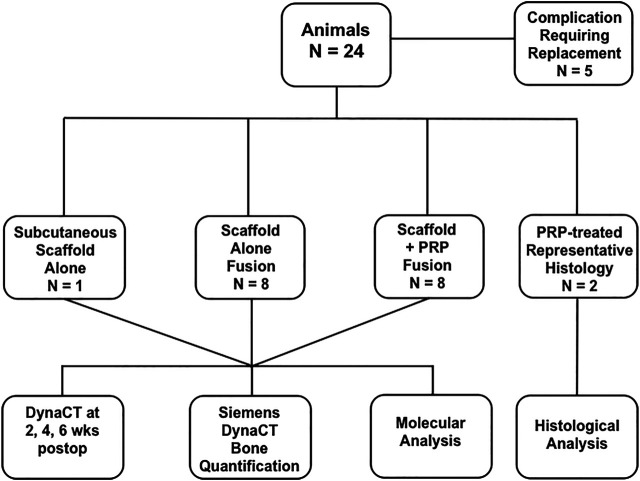
Study design.

Guidelines from the American Association for Laboratory Animal Science (AALAS) and the National Institute of Health (NIH) Guide for the Care and Use of Laboratory Animals and were strictly enforced for invasive animate procedures and all work was supervised and ethically approved by the Houston Methodist Research Institute (HMRI) Institutional Animal Care and Use Committee (IACUC, AUP-0115-002). Female New Zealand White rabbits (N = 8/group, Charles River Labs, Houston, TX) weighing an average of 3.8 kg were allowed at least 72 h of acclimation time upon arrival and were housed individually with free ambulation and food/water ad libitum before any invasive operation. Group 1 rabbits received our nanocomposite MHA/Coll scaffolds + PBS after decortication and Group 2 rabbits received nanocomposite scaffold + autologous PRP. To obtain baseline image density *in vivo* and prevent false positive quantification of novel bone deposition, one additional rabbit underwent subcutaneous placement of three scaffolds alone for 24 h prior to DynaCT imaging. In total, five rabbits required replacement in the study due to complication. Representative histology of untreated, implanted MHA/Coll scaffolds have previously been published (Minardi et al., 2019), so one animal from each group was allocated for representative histology, making a total of N = 24 rabbit subjects utilized in the study (Figure 1). A time point of 6 weeks postoperatively was chosen for euthanasia and specimen harvest. Although we recognize that further bone maturation occurs after this time, this should allow adequate time for an objective measure of novel collagen/osteoid deposition and evidence of scaffold remodeling. All 16 animals taken to the end of the study period had implanted specimens evaluated by molecular analysis.

### Orthotopic Surgical Model

The nanocomposite MHA/Coll scaffold utilized in this study has previously demonstrated osteoconductive potential by recapitulating a bone marrow-like 3-dimensional niche and has been exhaustively characterized (Minardi et al., 2015; Minardi et al., 2019). Here we applied PRP to evaluate the ability of autologous factors to augment bone regeneration within these osteoconductive scaffolds *via* a truly osteogenic remodeling process. To that end, we implanted MHA/Coll scaffolds unilaterally in an orthotopic model of single-level, posterolateral lumbar spinal fusion in similar fashion to established models described elsewhere (Boden et al., 1995; Morone and Boden, 1998). Ethylene oxide was used to sterilize MHA/Coll scaffolds (4 × 1 cm) prior to surgical implantation using an AN74ix chamber (Andersen, Haw River, North Carolina). Much work has been done previously using simplistic collagen scaffolds and osteoregenerative therapeutics, including PRP, some failing to show significant regenerative effect (Sarkar et al., 2006). Decortication is a known impetus to quicken healing and promote bone generation (Yamauchi et al., 2015). We elected to use a decortication-alone control on the anatomical left rather than simplistic collagen scaffold atop decortication.

**FIGURE 2:**
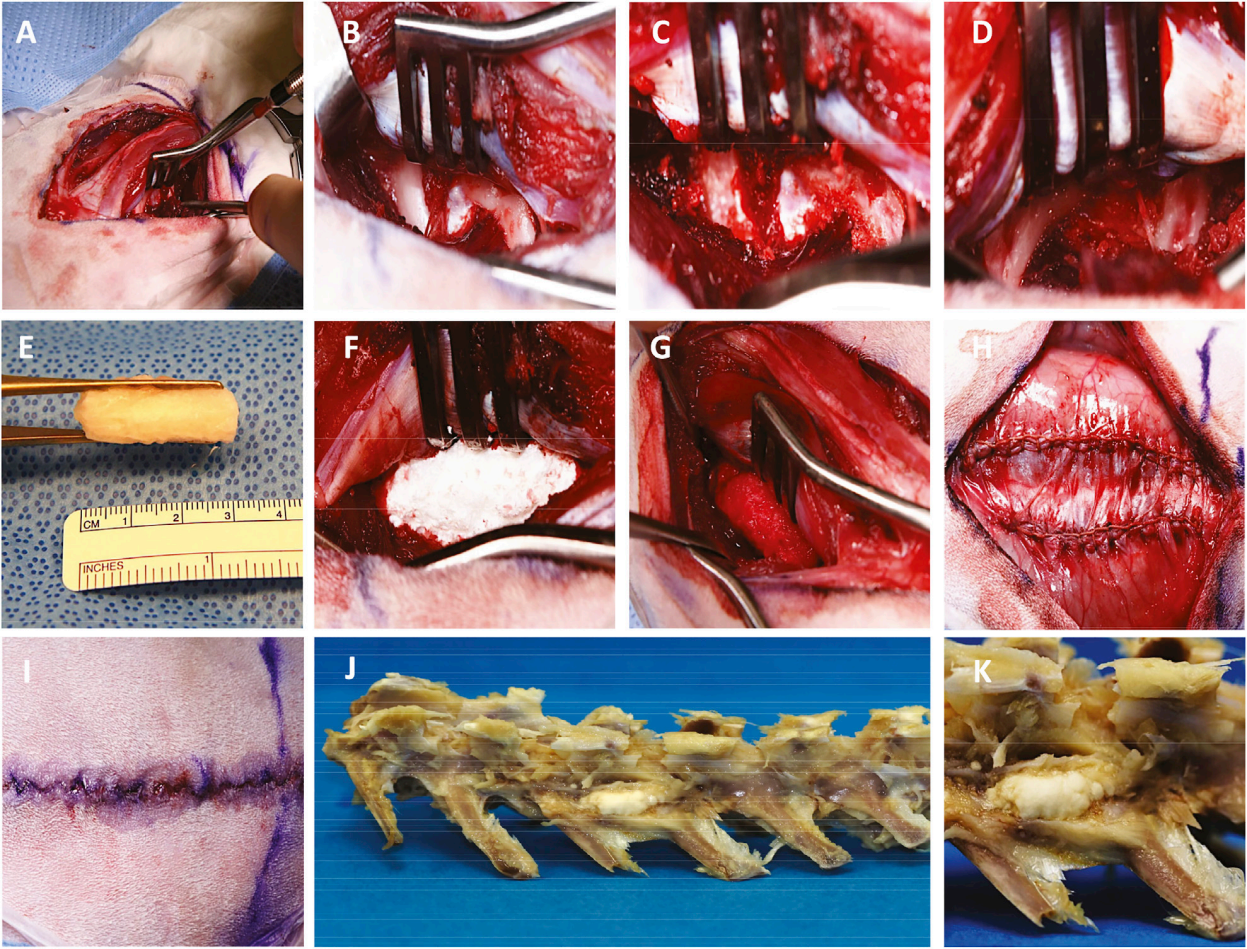
Operative technique of posterolateral lumbar spinal fusion. Dissection through the skin and intermuscular planes **(A)** exposes adjacent lumbar vertebral bodies and transverse processes **(B)** which are prepared by decortication **(C, D)** for fusion using a customized 3 cm nanocomposite MHA/Coll scaffold **(E)** either alone **(F)** or soaked in autologous PRP **(G)**. The tissues are closed with absorbable suture and skin glue **(H, I)** and harvested at 6 weeks postoperatively **(J, K)**.

We followed identically the surgical fusion procedure as previously reported (Minardi et al., 2019). While in the prone position, general anesthesia was provided by HMRI veterinary personnel using a combination of inhaled isoflurane anesthesia and intravenous ketamine and midazolam. Under sterile conditions, an 8 cm dorsal midline incision was made over adjacent lumbar spinous processes through the skin and subcutaneous tissues (Figure 2A) followed by bilateral 6 cm incisions lateral to the palpable mammary bodies from the L4-L7 vertebrae (Figure 2B). The transverse processes (TPs) of L5-L6 were exposed by mostly blunt dissection between the paraspinal (longissimus, multifidus, and ileocostalis) muscles (Figure 2C) and cleared using a periosteal elevator (Figures 2D,E) prior to decortication with a high-speed cone burr (Figure 2F) until punctate bleeding was visualized—a known stimulus for bone growth (Canto et al., 2008). The neurovascular bundle exiting the spinal foramen at the superior edge of the vertebral body (VB)-TP junction was carefully preserved. The anatomical left side served as an internal control, receiving decortication alone. The anatomical right side received a scaffold trimmed to 3 cm in length to bridge the adjacent decorticated TPs, soaked with an equivalent 2 ml dose of PBS or autologous PRP, depending on the experimental group. Incisions were approximated with absorbable suture, and after 6 weeks *in vivo*, animal subjects were euthanized by carbon dioxide inhalation, for harvest of the biomaterial samples. Under the effects of anesthesia under similar sterile conditions, a control animal received three subcutaneously implanted scaffolds in the dorsal tissue after creation of a subcutaneous pocket using blunt dissection in the supramuscular space through separate 3 cm incisions at least 5 cm apart from one another, approximated using an absorbable subcuticular suture and skin glue prior to CT and euthanasia 24 h later.

### DynaCT Imaging Analysis and New Bone Mass Quantification

To visualize and quantify new bone formation within scaffolds over time, advanced 3-dimensional (3D) computed tomography (DynaCT) imaging was utilized using a Siemens Axiom Artis C-arm (d)FC (Siemens Healthcare, Erlangen, Germany) scanner with a 48 cm × 36 cm flat-panel integrated detector under the following acquisition parameters: 70 kV tube voltage, automatic tube current of 107 mA, 20 s scan. With one image taken every 0.5 degrees of 270 total degrees of rotation, each acquisition generates 540 individual images for reconstruction. Lumbosacral DynaCT scans were obtained at 2, 4, and 6 weeks postoperatively with experimental animals lightly anesthetized using Midazolam (1 mg/kg) and inhaled isoflurane (2–3%). Individual scans were rendered for 3D reconstruction using proprietary Siemens software. After defining the surgical site region of interest, the density threshold of displayed tissues was manually set to that of bony structures, allowing automatic removal of all extraneous non-bony soft tissues from view (Supplementary Video S1, 2).

Raw Digital and Imaging Communications in Medicine (DICOM) files from the DynaCTs were loaded into Inveon Research Workplace 4.2 Software (Siemen Medical Solution, United States, Inc.) and identically-sized regions of interest (ROIs) that encompassed the implanted scaffold or the decorticated area (control) were manually selected for quantification of new bone growth according to established densitometric thresholds for trabecular (200 Hounsfield Units, HU) and cortical bone (500 HU) respectively (Rho et al., 1995; de Oliveira et al., 2008; Hartmann et al., 2010). New bone volume at either density was calculated according to the formula: New Bone Volume (mm^3^) = Experimental Side ROI—Control Side ROI. To both prevent a false positive postoperative interpretation and acquire an accurate baseline density measurement, saline-hydrated scaffolds were assessed extracorporeally and 24 h after subcutaneous implantation by DynaCT and Inveon Workplace quantification. Mean subcutaneous scaffold density at 200HU and 500HU was then subtracted from quantified values obtained in experimental subjects at 2, 4, and 6 weeks postoperatively.

### Clinical Assessment of Fusion

At the time of euthanasia at 6 weeks, success of fusion was assessed by a blinded orthopedic surgeon observer using manual palpation to test for segmental motion of the lumbar spine as previously reported (Miyazaki et al., 2008). Motion was expected on the anatomical left side decortication control, but any motion detected on the experimental right side between transverse processes was considered a failure of fusion. Successful fusion was signified by the absence of right-sided motion.

### Molecular Gene Expression Analysis

Implanted scaffolds were harvested at 6 weeks postoperatively for molecular expression analysis by RT-qPCR (reverse transcription quantitative polymerase chain reaction) compared with a control of native bone from rabbit transverse process. Extracted specimens were treated in 1 ml of Trizol reagent (Life Technologies) prior to homogenization and RNA extraction using RNeasy column (Qiagen) according to the manufacturer’s stated protocol. A NanoDrop ND1000 spectrophotometer (NanoDrop Technologies) was used to calculate the purity and total concentration of RNA present. Using 1,000 ng of total of RNA and the iScript retrotranscription kit (Bio-Rad Laboratories), we synthesized cDNA. Finally, we analyzed the transcribed cDNA product on a StepOne Plus real-time PCR system (Applied Biosystems) using Taqman^®^ fast advanced master mix and the probes (Thermo-Fisher Scientific) of interest signifying osteoblastic differentiation with Runt-related transcription factor 2 (RUNX2, Oc02386741_m1), and osteogenesis with bone matrix remodeling and mineralization with osteonectin (secreted protein acidic and rich in cysteine, SPARC, Oc03395840_m1) and osteopontin (secreted phosphoprotein 1, SPP1, Oc04096882_m1). We hypothesized that the microenvironment was sufficiently different along the length of the implanted scaffold (hypoxia, cell density, cellular cues, etc.) that it warranted investigating separately the cranial (Cr), middle (M), and caudal (Ca) portions of explanted PRP-treated samples in Group 2 for molecular expression. Mean relative-fold expression of the genes of interest was calculated for both groups compared to a transverse process bone control. To control for the effect of PRP treatment, expression in Group 1 was also directly compared to that of Group 2.

### Histomorphometry

All specimens were transported and received in ethanol solution. After additional minor tissue trimming to prepare the specimens for processing into methyl methacrylate (MMA, Sigma-Aldrich, St. Louis, MO) resin, specimens were post-fixed in 10% neutral buffered formalin (NBF) for 24–48 h at ambient temperature (RT) before transfer to a 70% ethanol solution while under gentle, constant agitation. Using an automated tissue processor (ASP300S, Leica, Germany), tissues were sequentially dehydrated with increasing ethanol concentration (70–100%) over several days. Specimens were transferred to 100% methyl salicylate (Sigma-Aldrich, St. Louis, MO) manually cycled over 48–72 h between gentle agitation and vacuum chamber, periodically observing tissues for translucence to confirm dehydration. Tissue clearing was completed with 100% xylenes (Sigma-Aldrich, St. Louis, MO) using the automated tissue processor. Specimens underwent resin infiltration using multiple exchanges of freshly prepared MMA and dibutyl phthalate (Sigma-Aldrich, St. Louis, MO) solution at RT over multiple days while cycling between gentle agitation and vacuum chamber. Samples were transferred to pre-polymerized base molds within sealable containers, where a fourth and final resin solution was added with a benzoyl peroxide-based catalyst to initiate a curing, exothermic polymerization reaction of each specimen into a clear MMA block over several days. Microtomy was performed at 5 microns using a motorized SM2500 sledge microtome (Leica, Germany) and d-profile (sledge) tungsten-carbide knives (Delaware Diamond Knives). Each individual 50 mm × 75 mm glass microscope slide (Fisherbrand) was coated with an in-house prepared gelatin-based solution and covered with a plastic protective strip and heated overnight at 55°C. Goldner’s Trichrome staining were used to visualize contrast between bone soft tissue morphology and differentiate newly formed bone, dense collagen, or osteoid from native mineralized bone. Metachromatic MacNeal’s Tetrachrome stain with a pre-staining Von Kossa reaction was used to demonstrate osteoclastic and osteoblastic activity laying down dense collagen and osteoid and standard hematoxylin and eosin (H and E) was used for cellular detail.

### Statistical Analysis

Statistical analysis was performed using GraphPad Prism Software (San Diego, CA, United States). All experiments were performed at least in triplicate (see individual paragraphs for specific number of replicates). A repeated-measures ANOVA (analysis of variance) was performed to compare new trabecular and cortical bone growth volume over time in Groups 1 and 2. Paired t-tests were done to compare differences in molecular expression of genes of interest using trabecular bone expression as control. A similar analysis was performed comparing Group 1 scaffold alone expression with Group 2 PRP-treated samples. A one-way ANOVA with Tukey’s multiple comparisons test was also performed for each gene of interest to evaluate for differences in expression amongst all groups including Group 2 subsets. In all cases, an alpha of 0.05 was used and significance was represented as follows: * was used for *p* < 0.05, ** for *p* < 0.01, *** for *p* < 0.001 and **** for *p* < 0.0001.

## Results

### Scaffold Characterization and Biomimicry

**FIGURE 3:**
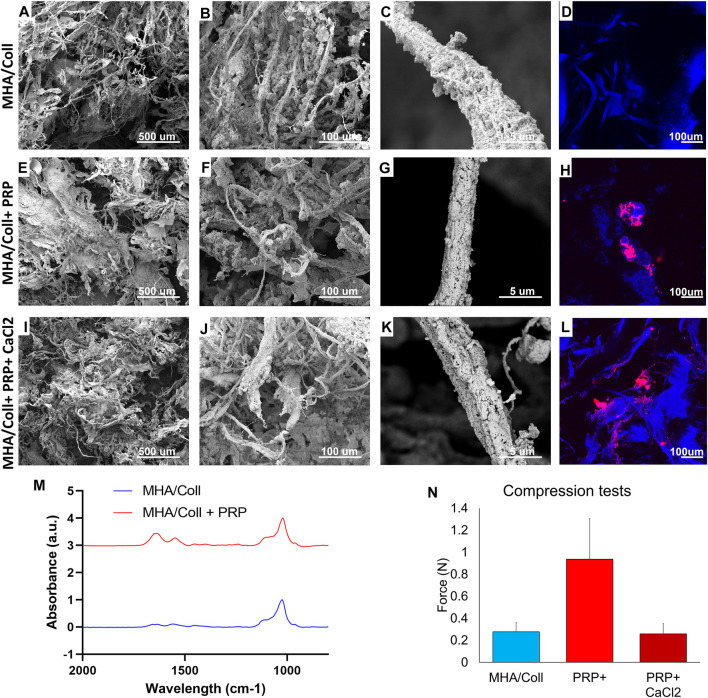
Material Characterization. SEM was used to characterize surface topography, pore size, and uniformity of our MHA/Coll scaffold alone **(A–C)**, MHA/Coll + PRP **(E–G)**, and MHA/Coll + PRP + CaCl2 **(I–K)**. Confocal microscopy was used in a DAPI channel to characterize collagen fibril autofluorescence including on MHA/Coll scaffolds alone **(D)**, and distribution of labeled PRP alone **(H)** or after CaCl2 activation **(L)**. FTIR analysis was used to assess differences in spectra from MHA/Coll scaffolds alone or with the addition of PRP **(M)**. Changes in compression/stretch were also characterized **(N)**.

MHA/Coll scaffolds alone have wide fibers completely filled and covered by hydroxyapatite nanoparticles causing a range of smaller, anisotropic pores (Figures 3A,B). Consistent with what we previously reported (Minardi et al., 2015; Minardi et al., 2019), the collagen fibers appeared fully and homogenously mineralized (Figures 3C,G,K). These fibers autofluoresce under confocal microscopy (Figure 3D). The addition of PRP did not significantly alter the topography of the scaffold material, with similar pore sizes and homogeneous fibril mineralization seen on SEM (Figures 3E–G). Platelets adhered to the surface of scaffold fibrils in variable-sized clusters on confocal imaging (Figure 3H). The addition of CaCl2 to activate PRP did not affect either the scaffold porosity/topography witnessed by SEM (Figures 3I–K), nor the distribution of PRP along fibrils (Figure 3L).

FTIR spectra reported in Figure 3M show the typical peaks of HA present in the area of 1000cm^−1^. The amide bands in the region 1,500–1700 cm^−1^ are more intense when the PRP (red line in Figure 3M) has been added to the scaffold confirming the presence of more proteins and cell components that increase the intensity of vibrational modes. Amide I (1,700–1,600 cm^−1^) and amide II (1,600–1,500 cm^−1^) are related to the stretching vibration of C = O bonds and to C–N stretching and N–H bending vibration, respectively—chemical components well known to be densely present in protein structure.

Lastly, compressive tests were carried out to evaluate the influence of PRP content on the strength and stiffness of the scaffold. Figure 3N summarizes the very weak resistance to compression observed as expected for spongy scaffolds, as has been previously demonstrated for this type of work (Ghodbane and Dunn, 2016). In comparison with the empty baseline scaffold (0.278N ± 0.082), the mean compression resistance significantly increased in the presence of native PRP without CaCl2 (0.937N ± 0.369). However, that difference in mean compression resistance disappears when PRP activated by calcium chloride (0.259N ± 0.095) is used in similar fashion to what was used in our *in vivo* model, perhaps due to platelet lysis and or release of its alpha granule cargo, effectively changing the composition of the PRP. This is a phenomenon that requires further study.

### Operative Outcomes

MHA/Coll scaffolds and autologous PRP were easy to handle and customize operatively and the PRP was consistently adsorbed fully by the scaffold. Rabbits are notoriously fragile surgical subjects, particularly when general endotracheal anesthesia is required. Five rabbits in total suffered complications of surgery requiring replacement in the study—four died from respiratory complications of general anesthesia and one rabbit was humanely euthanized at the 2-weeks time point due to surgical site infection.

### Clinical Assessment of Fusion

Both experimental groups formed prominent, hardened growth at the surgical site of implantation, but Group 2 growth was consistently more robust. PRP-treated Group 2 rabbits displayed a superior clinical rate of fusion at 75% (6/8 animals) compared to 25% (2/8 animals) in Group 1. These findings correlated well with radiographic data with an overall rate of accuracy of 93%.

### DynaCT 3D Imaging

**FIGURE 4:**
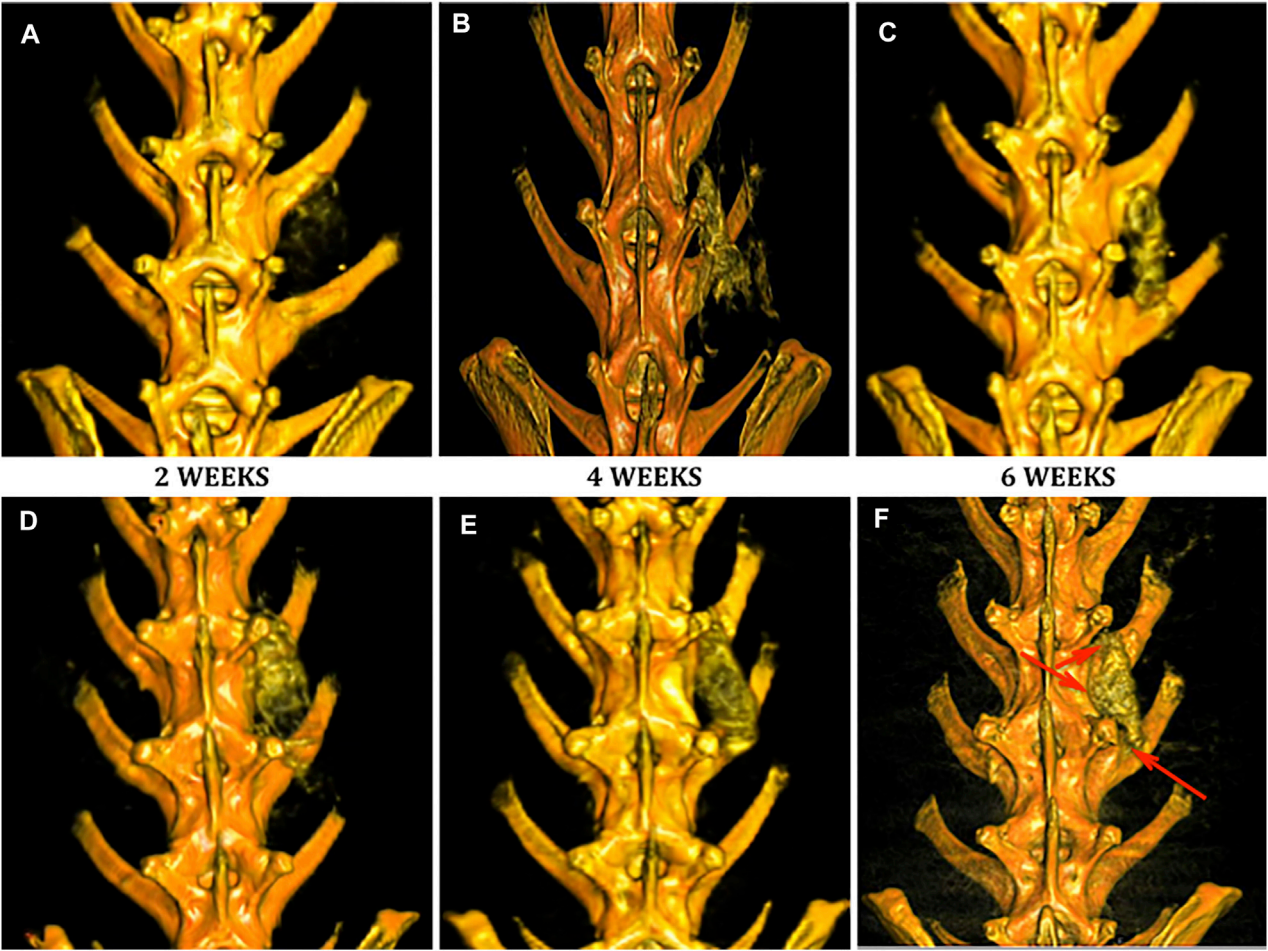
Evaluation of fusion by DynaCT with 3D reconstruction. Representative 3D reconstructions of spinal DynaCT are shown at 2, 4, and 6 weeks for Group 1 nanocomposite scaffold alone **(A–C)** and Group 2 PRP-treated **(D–F)**. Areas of increased bone growth and fusion were seen at 6 weeks most prominently in Group 2 specimens (arrowheads).

**FIGURE 5:**
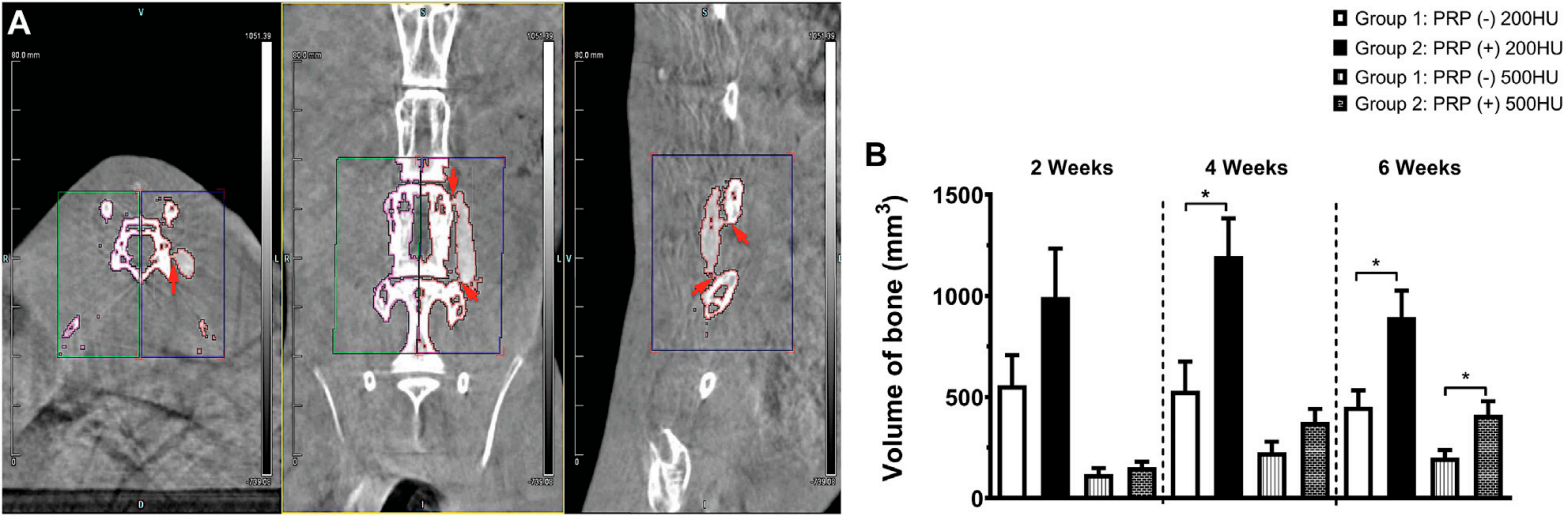
Volumetric quantification of osteogenesis. Areas of bridging bone and fusion (arrowheads) were clearly appreciated on axial, coronal, and sagittal CT views where identically sized ROI’s were selected **(A)** on the experimental right and control left sides for volumetric new bone quantification. Newly formed trabecular (200HU) and cortical (500HU) bone was quantified and compared between the two experimental groups over time **(B)**, *p < 0.05.

No implanted scaffolds migrated from the surgical site and all remained in direct contact with adjacent decorticated transverse processes. Both Group 1 (Figures 4A–C) and Group 2 (Figures 4D–F) specimens displayed increased bony density and prominence of the implanted scaffold over the time period of the study as expected. By 6 weeks, both groups demonstrated some degree of bridging bony remodeling of the nanocomposite scaffold approaching native spinal bone density albeit with variable homogeneity. CT imaging confirmed fusion in 1/8 (12.5%) of Group 1 animals—slightly less than predicted by clinical assessment. However, PRP-treated Group 2 scaffolds consistently showed earlier, more homogeneous and a larger amount of bony replacement compared to Group 1 with bridging bone formation in 100%, and fusion confirmed in 6/8 animals (Figure 4, Figure 5A).

### Quantitative Analysis of New Bone Volume

As expected, with a density slightly higher than that of air, extracorporeal scaffolds displayed no quantifiable volume at the requisite densities. At 24 h post-implantation, the average volume of a hydrated scaffold at 200HU threshold was 69 mm^3^ (± 9.8), which was used as baseline and all subsequent measurements had this value subtracted from the total. As expected, the volume at 500HU threshold at 24 h was zero.

The ROI volume captured was identical on the anatomic left and right of each animal (Figure 5A). Group 1 animals generated a significant amount of novel bone over time compared to controls, but Group 2 animals produced more of both trabecular (200HU) and cortical (500HU) bone at all time points than Group 1 animals (Figure 5B). At 2 weeks, this increase trended toward but did not reach statistical significance. By 4 weeks, PRP-treatment produced significantly higher mean trabecular bone volume than scaffold alone Group 1 specimens (1,196.3 versus 531.6mm^3^, *p = 0.0295). At 6 weeks, PRP treatment generated significantly more of both trabecular (895.6 versus 453.2 mm^3^, *p = 0.020) and cortical bone (412.3 versus 200.6 mm^3^, *p = 0.027). Repeated measures ANOVA demonstrated significant differences between groups for both 200HU trabecular bone (*p = 0.045) and 500HU cortical bone (**p = 0.0034).

### Molecular Analysis

The ability of the MHA/Coll nanocomposite scaffold to promote osteogenic gene upregulation both *in vitro* and *in vivo* has previously been established (Minardi et al., 2019). Both Group 1 and Group 2 specimens using the nanocomposite scaffold displayed significant upregulation of all three genes of interest as compared to expression in native bone control. Group 2 animals consistently displayed the highest relative fold expression and generally speaking, expression was higher at the terminal cranial and caudal ends of the implanted scaffold than the mid portion.

**FIGURE 6:**
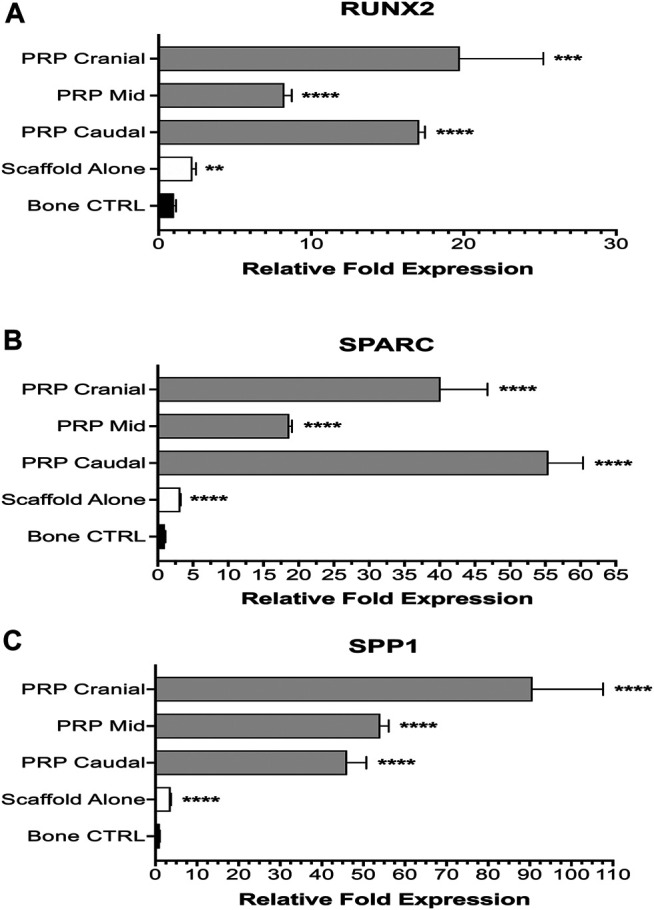
Molecular analysis of osteogenesis. Group 1 specimens were compared with separate cranial, mid, and caudal regions of Group 2 specimens for expression of osteogenic genes: RUNX2 **(A)**, SPARC **(B)**, and SPP1 **(C)**.

For RUNX2, both experimental groups displayed significant upregulation compared to vertebral bone control (Figure 6A). Group 1 animals averaged 2.21-fold higher expression (± 0.24, ****p < 0.0001). PRP-treated Group 2 animals had even higher expression—cranial (19.74-fold ± 5.48, ***p = 0.0002), mid (8.23-fold ± 0.50, ****p < 0.0001), caudal (17.08-fold ± 0.38, ****p < 0.0001). Group 2 animals also had significantly higher upregulation when compared to Group 1 at all three subsites (****p < 0.0001). Expression at cranial and caudal sites was not significantly different from one another but both showed significant upregulation compared to the mid portion of the PRP-treated scaffold (****p < 0.0001). A one-way ANOVA also demonstrated a significant difference among all subgroups (F = 57.87, ****p < 0.0001).

The degree of upregulation was even more striking for SPARC (Figure 6B). Compared to bone control, Group 1 animals averaged 3.20-fold higher expression (± 0.07, ****p < 0.0001). All three subsites in Group 2 displayed very significant (****p < 0.0001) upregulated expression—cranial (40.14-fold ± 6.66), mid (18.67-fold ± 0.37) and caudal (55.44-fold ± 4.90). Once again, Group 2 animals had significantly higher expression compared to scaffold alone Group 1 animals as well at all three subsites (****p < 0.0001). A significant difference was also seen on one-way ANOVA among all subgroups (F = 213.7, ****p < 0.0001).

Lastly, for SPP1 all experimental groups displayed significant upregulation (****p < 0.0001) compared to bone control—Group 1 (3.67-fold ± 0.09), Group 2 cranial (90.65-fold ± 16.97), mid (54.04-fold ± 2.03), and caudal (46.09-fold ± 4.60) (Figure 6C). One-way ANOVA exhibited significant differences (F = 107.2, ****p < 0.0001) amongst groups. Within Group 2 subgroups, PRP cranial had significantly higher (****p < 0.0001) expression than both PRP mid and caudal subgroups, which had a non-significant difference from one another.

### Bone Histomorphometry

**FIGURE 7:**
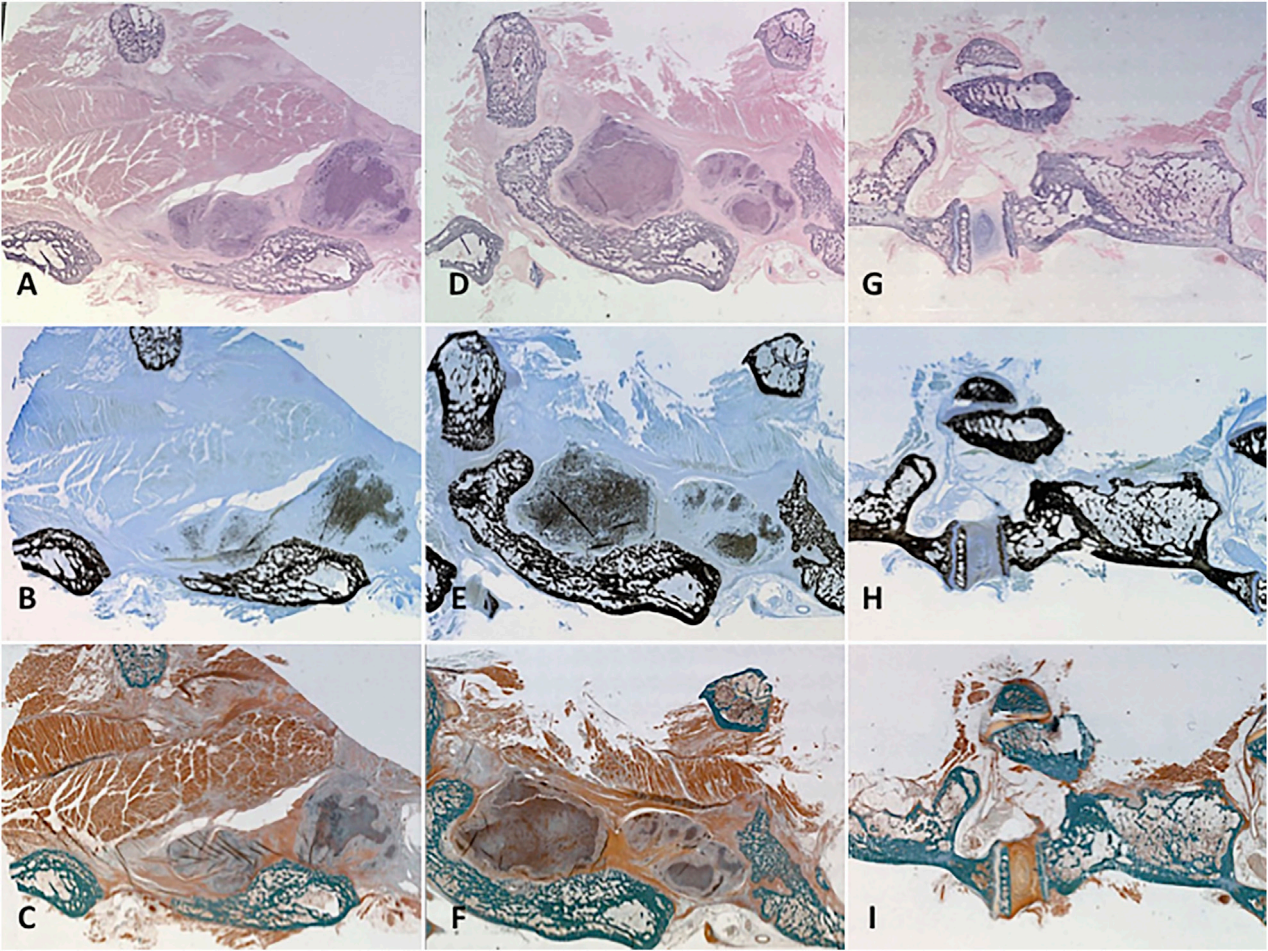
Histology. Representative specimens were evaluated for cellularity and mineralized tissue using three different stains. Cellularization of the remodelled scaffold with osteoblasts/clasts was signalled by pronounced hematoxylin staining of the treated scaffold on H and E stain to variable degrees according to treatment group **(A, D, G)**. Mineralized osteoid within the scaffold showed itself as “peppering” similar to native bone on Von Kossa-MacNeal’s Tetrachrome stain **(B, E, H)** and similar jade green appearance to native bone on Goldner’s Trichrome stain **(C, F, I)**. Group 1 **(A–C)** displayed significant osteoblast recruitment and mineralized osteoid production, but not as much as PRP-treated Group 2 **(D–F)**. A native control vertebral body-transverse process junction is shown **(G–I)** for reference.

Our prior work demonstrated the osteoconductive properties of MHA/Coll scaffolds when implanted alone in this orthotopic model, promoting scaffold population by osteoblasts and osteoclasts along with generation of mineralized bone matrix and osteoid that closely mimics native trabecular bone (Minardi et al., 2019). On H and E stain, higher density of nuclear material within osteoblasts, osteoclasts and osteocytes makes for a more blue appearance to native and newly formed bone as compared to the heavier cytoplasmic and further spaced nuclei of the more pink surrounding soft tissue and muscle. Implanted PRP-treated scaffolds maintained their close apposition to decorticated bone and displayed a similar degree of hematoxylin staining to native bone due to dense osteogenic cellular infiltration and nuclear stain uptake (Figures 7A,D). MacNeal’s Tetrachrome is a metachromatic stain that highlights cellularity between osteoclastic bone resorbing cells and osteoblasts actively laying down dense collagen to become mineralized trabecular bone (osteoid). This is seen as a black dot or peppering atop a gray-blue background signifying dense collagen/osteoid becoming mineralized as it is integrated with the mature/native trabecular bone, which has a characteristic jet black color appearance from the binding of silver ions to calcium in a pre-staining Von Kossa reaction before MacNeal’s metachromatic counterstain. Serial sectioning demonstrated substantial “peppering” of the PRP-treated scaffold from deposition of osteoid and mineralized bone content (Figures 7B,E). Goldner’s Trichrome staining (Figures 7C,F,I) was used to visualize contrast between bone soft tissue morphology and identify newly formed bone/dense collagen/osteoid (red) as compared to existing mature/native mineralized bone (jade green). Group 1 specimens (Figures 7A–C) displayed an ability to recruit osteoblasts and form dense collagen and osteoid within a remodeled scaffold alone, but not as prominently as Group 2 animals. Group 2 specimens prominently exhibited a greater mixture of osteoid and dense collagen within the remodeled scaffold (Figures 7D–F) and areas of particularly prominent mineralized bony content at areas of fusion with vertebral bone (Figures 7E,F).

## Discussion

Surgical fusion of the spine remains a fundamental procedure for the aging and trauma population and one primed for improvements by way of biomedical innovation for regenerative bone tissue engineering. Such innovation can come through improved biomaterial, cell-based, or extrinsic bioactive molecule moieties. This study provides valuable *in vivo* evidence that bone regeneration and spinal fusion is possible using an advanced nanocomposite scaffold and PRP as a source of autologous bioactive factors. The biocompatible, osteoconductive nature of our nanocomposite MHA/Coll scaffold was confirmed, and its moderate osteoinductive activity was significantly enhanced by the addition of autologous PRP, sufficient to induce clinical fusion. This was exemplified by both groups forming trabecular/cortical bone within a remodeled scaffold over 6 weeks, both displaying significant upregulation of key osteogenic genes above a native bone control, and both showing capability to induce clinical and/or radiographic bony fusion. Histomorphometry demonstrated significant osteoid population/replacement of the scaffold without significant immune degradation or foreign body reaction. Compared to Group 1 animals, Group 2 animals treated with PRP regenerated significantly more bone at earlier time points, more mature cortical bone at 6 weeks, displayed significantly higher gene upregulation, and a higher rate of both clinical and radiographic fusion. One can conclude from the results that the optimum implantable combination for bone regrowth should include a mineralized, nanocomposite scaffold with a hierarchical structure of biomimicry and bioactive molecules such as those delivered within autologous PRP.

The biocompatibility of our MHA/Coll scaffold is congruent with existing studies using collagen-based scaffolds (Asghari et al., 2017). Some of the enhanced cellular effects witnessed in our study can be explained by what we know about the effect of structural nanocomposition in combination with matrix components, such as those from PRP, translating to substantial regenerative tissue effects. According to Christy et al., the addition of hydroxyapatite nanocrystals is known to enhance vascularization and osteogenesis, while the freeze-drying process improves bioactivity and mineralization (Christy et al., 2020). Fibrin, obtained from autologous plasma sources like PRP, is a natural bioactive scaffold with several advantages over other tissue engineering moieties besides its hemostasis, biocompatibility and biodegradability. Numerous protein interaction sites on fibrin facilitate improved cellular proliferation, differentiation and growth factor expression, resulting in enhanced angiogenesis and wound healing (Noori et al., 2017; Christy et al., 2020). Platelet-rich fibrin (PRF) or PRP-fibrin applications can stimulate stem cell osteogenic differentiation, increase bone cure rates, and are sufficient to heal rabbit calvarial bone defects (Ahmed et al., 2008; Lalegul-Ulker et al., 2019; Christy et al., 2020). The fibrin-rich, bioactive matrix provided by PRP in Group 2 animals may explain the osteogenic cellularization, robust bone regeneration, and enhanced fusion outcomes witnessed in our study.

These results must be evaluated within the context of a conflicting background of prior literature on PRP’s ability to augment spinal fusion when applied with various regenerative constructs. For example, Li et al. studied fusion in a porcine model using a carbon fiber cage and beta tricalcium phosphate (β-TCP) with or without PRP compared to iliac crest autograft (Li et al., 2004). They witnessed only partial fusion in animals treated by β-TCP with no difference in the PRP group. The early experience of our senior author did not demonstrate improved fusion when PRP was used with autologous ICBG (Weiner and Walker, 2003). In contrast, a recent randomized controlled trial demonstrated improved fusion with significantly higher bone mass and greater bone union with the addition of PRP (Kubota et al., 2019). Liu et al. report 100% fusion in a rabbit model when using PRP embedded within a sheet of bone marrow-derived MSC’s (BMSC) compared to 0% fusion with BMSC alone, and 83% fusion using an ICBG (Liu et al., 2017). These two applications are very unique from one another in their macro/microstructure and mechanisms of action. We know that detailed elements of scaffold design ranging from composite elements, to porosity, to intrafibrillar versus extrafibrillar mineralization technique can have huge implications for bone regenerative efficacy (Uskoković, 2015). It follows then, that the ability for bioactive factors applied in concert with biomaterials to augment bone regeneration could be dramatically affected by dose or method of application (e.g., within scaffold matrix versus topical exogenous application, burst versus timed release, etc.). Recent studies particularly employing the therapeutic combination of PRP and composite biomaterials using porous collagen and apatite have consistently shown a beneficial effect with PRP addition (Oryan et al., 2012; Nosrat et al., 2019). PRP may have a narrower spectrum of biologic materials capable of exploiting its beneficial therapeutic effect when combined, but regardless, the mechanisms behind this inconsistent benefit warrant further study.

Despite the valuable implications of the results of this study, some inherent limitations exist. Although the decortication model has been used widely in published studies as a bone-forming control, in retrospect, using a simplistic, non-functionalized, collagen scaffold without hierarchical structure as a left-sided internal control would have enhanced the power of our results and will be utilized in future studies. Also, although using advanced CT imaging is certainly diagnostic for declaring fusion, quantitative bone histomorphometry is used more commonly in the literature for bone regeneration. Explanted rabbit spines were too large for processing by classic *ex vivo* quantitative bone histomorphometry, so we will consider using a rodent model in future studies to facilitate use of this modality as previously published (Zhu et al., 2004). Rodents are also a heartier species and their use may avoid the unnecessary morbidity/mortality from anesthesia complications witnessed in our rabbit model. Cost constraints limited the number of histologic specimens we could process only to representative experimental subjects. Despite evidence now of PRP’s efficacy in multiple orthotopic models in multiple species, the ultimate translatability to human patients remains unknown until additional data is obtained from human subjects.

In conclusion, this study provides valuable *in vivo* feasibility evidence for bone regeneration using a tissue-engineered platform that is, not dependent upon stem cell-based techniques or supratherapeutic doses of an extrinsic growth factor classically employed. These techniques can be both financially limiting or fizzle out during a burdensome clinical approval process. It also provides helpful data on the cell and molecular effects at the intersection of native tissue and an implanted, biohybrid scaffold. In the disputed argument regarding the utility of PRP in regenerative bony applications, this study adds evidence for a beneficial effect and a biomimicry approach to scaffold design. The evidence herein is insufficient to change current spinal surgical practice or standard of care and well-designed, human clinical trials are required to further investigate. With such sizable osteogenesis occurring with only a well-designed, bioresorbable scaffold, and autologous factors, the results suggest that a reliable bone-forming therapeutic capable of nearly 100% fusion success could be feasible by combining our platform with a modest dose of exogenous bioactive molecule, such as BMP2—far less than previously used and well within the therapeutic window of clinical safety. This represents a particular area of subsequent interest for further study.

## Data Availability

The authors acknowledge that the data presented in this study must be deposited and made publicly available in an acceptable repository, prior to publication. Frontiers cannot accept an article that does not adhere to our open data policies.
